# Identification of hub genes and key pathways in arsenic-treated rice (*Oryza sativa* L.) based on 9 topological analysis methods of CytoHubba

**DOI:** 10.1265/ehpm.24-00095

**Published:** 2024-08-07

**Authors:** Zhen Yu, Rongxuan Wang, Tian Dai, Yuan Guo, Zanxuan Tian, Yuanyuan Zhu, Juan Chen, Yongjian Yu

**Affiliations:** 1 School of Grain Science and Technology, Jiangsu University of Science and Technology, Zhenjiang 212100, Jiangsu, China; 2 School of Food and Biological Engineering, Jiangsu University, Zhenjiang 212013, Jiangsu, China; 3 Jiangsu Provincial Engineering Research Center of Grain Bioprocessing, Zhenjiang 212100, Jiangsu, China; 4 College of Food Science and Engineering, Moutai Institute, Renhuai 564501, Guizhou, China

**Keywords:** Arsenic, Rice, Bioinformatics analysis, Differentially expressed genes, CytoHubba

## Abstract

**Background:**

Arsenic is a toxic metalloid that can cause acute and chronic adverse health problems. Unfortunately, rice, the primary staple food for more than half of the world’s population, is generally regarded as a typical arsenic-accumulating crop plant. Evidence indicates that arsenic stress can influence the growth and development of the rice plant, and lead to high concentrations of arsenic in rice grain. But the underlying mechanisms remain unclear.

**Methods:**

In the present research, the possible molecules and pathways involved in rice roots in response to arsenic stress were explored using bioinformatics methods. Datasets that involving arsenic-treated rice root and the “study type” that was restricted to “Expression profiling by array” were selected and downloaded from Gene Expression Omnibus (GEO) database. Differentially expressed genes (DEGs) between the arsenic-treated group and the control group were obtained using the online web tool GEO2R. Gene Ontology (GO) function and Kyoto Encyclopedia of Genes and Genomes (KEGG) pathway enrichment analysis were performed to investigate the functions of DEGs. The protein-protein interactions (PPI) network and the molecular complex detection algorithm (MCODE) of DEGs were analyzed using STRING and Cystoscope, respectively. Important nodes and hub genes in the PPI network were predicted and explored using the Cytoscape-cytoHubba plug-in.

**Results:**

Two datasets, GSE25206 and GSE71492, were downloaded from Gene Expression Omnibus (GEO) database. Eighty common DEGs from the two datasets, including sixty-three up-regulated and seventeen down-regulated genes, were then selected. After functional enrichment analysis, these common DEGs were enriched mainly in 10 GO items, including glutathione transferase activity, glutathione metabolic process, toxin catabolic process, and 7 KEGG pathways related to metabolism. After PPI network and MCODE analysis, 49 nodes from the DEGs PPI network were identified, filtering two significant modules. Next, the Cytoscape-cytoHubba plug-in was used to predict important nodes and hub genes. Finally, five genes [*Os01g0644000*, *PRDX6* (*Os07g0638400*), *PRX112* (*Os07g0677300*), *ENO1*(*Os06g0136600*), *LOGL9* (*Os09g0547500*)] were verified and could serve as the best candidates associated with rice root in response to arsenic stress.

**Conclusions:**

In summary, we elucidated the potential pathways and genes in rice root in response to arsenic stress through a comprehensive bioinformatics analysis.

## 1. Introduction

Arsenic, which can be divided into inorganic and organic arsenic, is a toxic metalloid that can cause numerous adverse health problems such as skin damage [1], lung oxidative stress and inflammation [2], diabetes mellitus [3] and the neurological symptoms [4], and might be related to some endemic diseases [5]. Moreover, chronic exposure to arsenic can cause many cancers, such as colorectal cancer [6], lung cancer [7, 8], bladder cancer [9], multiple cutaneous carcinoma [10]. Arsenic and its compounds had been identified as Class I carcinogens by the International Agency for Research on Cancer (IARC). It is estimated that the groundwater of nearly 108 countries worldwide is contaminated by arsenic and more than 230 million people, including 180 million from Asia, are at risk of arsenic poisoning [11]. Besides, more than two million people worldwide are known so far to have been affected by arsenic-related diseases [12]. Arsenic has received widespread attention as a result of its extreme toxicity and carcinogenicity.

Humans are exposed to arsenic in many ways, such as water, air, and the consumption of foods containing arsenic. Rice is the primary staple food for more than half of the world’s population. Unfortunately, rice is generally regarded as a typical arsenic-accumulating crop plant with a mean range of total arsenic concentration in brown rice grains was 0.17 to 0.45 mg kg^−1^ [13]. Arsenic stress could significantly impact rice chlorophyll and root parameters, reduce rice growth and grain yield [14, 15]. In order to reduce the accumulation of arsenic in rice grains, many practices have been researched when rice applied in hydroponic conditions, such as nutrients and fertilizers management, passivator addition, microbial supplementation and genetic engineering methods. By reducing the bioavailability of arsenic, the management of nutrients and fertilizers, and the addition of passivator could reduce the accumulation of arsenic in rice. But, the management of nutrients and fertilizers, including the addition of selenium, silicon [16], micronized zero-valent iron, sulfate, nitrate and plant hormones (melatonin, 2,4-epibrassinolide, jasmonic acid) [17–19], existed the problems of poor efficiency and high cost. The addition of passivator, including nanoparticles (zinc oxide, magnesium oxide, iron oxide, titanium dioxide) [20–23] and biochar [21], may pose secondary contamination risks [24]. Microbial supplementation is a relatively green approach. Some microorganisms could stabilize or dissolute arsenic and activate rice defense mechanisms, such as *Serratia marcescens* [25] and rhizobacteria [26]. In order to be applied to actual production, more microbial strains that could decrease arsenic content in rice are needed, but screening microbial strains require a significant amount of manpower and material resources. Obtaining rice varieties with high arsenic tolerance and low accumulation by genetic engineering methods is a sustainable and effective way to reduce arsenic accumulation in rice. But the major objective is to obtain rice genes related to arsenic stress. At present, several arsenic-related genes in rice have been found. For example, the phytochelatin synthase OsPCS1 have been identified to act as a crucial role in reducing arsenic levels in rice grains by sequestering phytochelatin-arsenic complexes into the vacuole [27]. Report showed that co-overexpression of gene *OsPCS1*(*Os05g0415200*) and the tonoplast transporter genes *OsHMA3*(*Os07g0232900*), *OsABCC1*(*Os04g0620000*) in rice could decrease arsenic concentration in grain by 92.1% [28]. In addition, the increase of the expression of NAC transcription factor gene *SNAC3*(*Os01g09550*) could enhance the tolerance of rice to arsenic stress through modulating antioxidants, photosynthesis, osmolyte accumulation, and stress-related genes expression [29]. Besides, the MYB transcription factor encoded gene *Os04g50680*, expressed almost exclusively in the rice root, might be also related to arsenic accumulation in rice for the up-regulated in the low-As-accumulation lines compared to the high-As-accumulation lines after As treatment [30]. However, more and more arsenic-related genes need to be found to meet the needs of different varieties of rice and growing environments.

Due to the development of molecular techniques, such as high-throughput sequencing and microarray, the genetic performance in rice responses to arsenic stress could be explained. Rice plants absorb arsenic mainly through the root. Therefore, in the present study, datasets related to arsenic-treated rice root and “study type” was restricted to “Expression profiling by array” were selected and downloaded. Differentially expressed genes (DEGs) that might play crucial roles in rice response to arsenic stress were screened out. The functions and roles of selected candidate DEGs in the arsenic-treated rice plant were then further evaluated.

## 2. Methods and materials

### 2.1 Expression profiles datasets

The gene chips related to arsenic stress in rice were screened out using the GEO database (https://www.ncbi.nlm.nih.gov/geo/). Three gene chips GSE25206, GSE99083, and GSE71492 were retrieved and downloaded, and the three gene chips were analyzed. Then, GSE99083 was excluded since it showed the response of rice leaves to arsenic stress. Finally, two expression profiles, GSE25206 and GSE71492, that showed the effects of arsenic stress on rice roots and both contained the experimental group and the control group, were selected for further analysis.

### 2.2 Differentially expressed genes identification

The online web tool, GEO2R (https://www.ncbi.nlm.nih.gov/geo/geo2r/), is often used to explore the genetic variations between two or more groups of samples based on the GEOquery and Limma R packages [31]. In order to obtain DEGs between the arsenic-treated group and the control group, two selected expression profiles, GSE25206 and GSE71492, were analyzed using the GEO2R tool and the genes that met the cut-off criteria of P-value <0.05 and |log fold-change| >1.0 were defined as DEGs.

### 2.3 Screening of common differentially expressed genes

After obtaining DEGs between the arsenic-treated group and the control group in both GSE25206 and GSE71492, the intersection of DEGs was acquired using Venn analysis [32] to screen out common DEGs between the above two expression profiles.

### 2.4 Functional and pathway enrichment analysis

In order to investigate the functions of the common DEGs originated from GSE25206 and GSE71492, Gene ontology (GO) function enrichment analysis and Kyoto Encyclopedia of Genes and Genomes (KEGG) pathway enrichment analysis, were performed by using the DAVID database [33] (Database for Annotation, Visualization and Integrated Discovery, https://david.ncifcrf.gov/). GO categorizes gene functions into three major categories: Biological process (BP), cellular component (CC) and molecular function (MF). A P-value of less than 0.05 was considered to be statistically significant.

### 2.5 Protein–protein interaction (PPI) network construction and hub gene analysis

In order to analysis the interactive relationships between DEGs, DEGs were mapped to STRING (Search Tool for the Retrieval of Interacting Genes/Proteins, https://cn.string-db.org/), and the experimentally validated interactions with a combined score of more than 0.4 were defined as significant. Then, PPI networks were visualized using Cytoscape 3.9.1. Subsequently, the molecular complex detection (MCODE) plug-in was used to filter the crucial modules in the PPI network using default settings (Degree cut-off = 2, Node score cut-off = 0.2, *K*-core = 2, Maximum depth = 100). DAVID was applied to analyze GO function and KEGG pathway enrichment of the identified modules. In addition, CytoHubba, a Cytoscape plug-in that provides 11 topological analysis methods, was used to predicate and explore the important nodes and hub genes in the PPI network. The top 10 genes of the results of topology analysis algorithms in CytoHubba were screened out and the final top 5 hub genes of the topology analysis algorithms were regarded as the best candidates associated with rice root in response to arsenic stress.

## 3. Results

### 3.1 Screening of DEGs from the gene expression profiles

Three gene chips GSE25206, GSE71492, and GSE99083 were retrieved and downloaded from GEO database. In GSE252006, the rice variety IR-64 germinated seeds were transferred to Hewitt solution for 10 d of growth. Then, seedlings were divided into the control group and the experimental group [34]. In the experimental group, seedlings were treated with 100 µM of pentavalent arsenic for 24 h. Total RNA was extracted from the treated rice roots and Affymetrix Gene Chip Rice Genome Arrays (Gene Expression Omnibus platform accession no. GPL2025) were used for microarray analysis. In GSE71492, the rice variety IR-64 germinated seeds were transferred to modified Yoshida medium for 15 d of growth. Afterwards, three-leaf stage seedlings were exposed to 25 µM of trivalent arsenic for 24 h. The control group were grown under normal condition without arsenic stress. Total RNA of the treated rice roots was extracted and GPL20752 platform were used for microarray analysis [35]. In GSE99083, Seeds of the rice cultivar Nipponbare were transferred to sandy loam soil and grown for four weeks [36]. Then, seedlings were subjected to 10 mM trivalent arsenic solution and 50 mM pentavalent arsenic solution for 7 d. Total RNA of the uppermost rice leaf blades was extracted and the mRNA expression was analyzed on Agilent-015241 Rice Gene Expression 4x44K Microarray. GSE99083 was excluded for the different rice variety and it showed the response of rice leaves to arsenic stress. Finally, two gene expression profiles related to arsenic-treated rice roots, GSE25206 and GSE71492, were selected for further analysis. Samples in the GSE25206 (three arsenic-treated samples, three control samples) and GSE71492 (two arsenic-treated samples, two control samples) were classified into two groups (arsenic-treated group and control group) and the DEGs were retrieved from the comparison of the two groups, respectively. Results showed that 173 up-regulated and 203 down-regulated genes were screened out from GSE25206, and 2152 up-regulated and 828 down-regulated genes were screened out from GSE71492. Both the top ten up-regulated and down-regulated DEGs in the two gene expression profiles are listed in Table 1.

**Table 1:** Identified DEGs in GSE25206 and GSE71492 (Top ten, arsenic-treated group versus control group).

**GSE25206**				**GSE71492**			

**ID**	**Gene symble**	**Fold change**	**P value**	**Gene ID**	**Gene name**	**Fold change**	**P value**
Down-regulated				Down-regulated			
Os.52208.1.S1_at	LOC4351146	−4.23	5.59E-07	4328052	Os02g0112100	−8.95	1.42E-06
Os.49146.1.S1_at	LOC4339786	−3.59	8.03E-05	9267850	Os12g0555100	−8.23	1.31E-06
Os.12340.1.S1_at	LOC4325264	−3.12	7.03E-06	4326278	Os01g0355250	−8.17	1.64E-06
Os.12313.1.S1_at	LOC4351694	−2.82	6.51E-06	4325264	Os01g0216000	−7.71	2.18E-06
Os.8014.1.S1_at	LOC4348209	−2.82	2.41E-04	4329797	Os02g0582900	−7.62	2.00E-06
Os.39038.1.A1_at	LOC4351966	−2.75	1.88E-04	4325264	Os01g0216000	−7.53	6.45E-07
Os.53276.1.S1_at	LOC4341340	−2.68	1.01E-04	4350344	Os11g0306400	−7.02	3.42E-05
Os.12296.1.S1_at	LOC4333359	−2.67	2.25E-05	4338119	Os05g0217800	−6.85	9.85E-06
Os.1314.1.S1_at	LOC4344439	−2.53	1.85E-05	4349245	Os10g0537800	−6.83	3.01E-06
Os.10959.1.S1_at	LOC4352021	−2.41	1.18E-06	4350388	Os11g0428800	−6.77	1.60E-06
Up-regulated				Up-regulated			
Os.9013.1.S1_at	LOC4349181	5.09	1.95E-05	4345814	Os08g0473900	12.78	2.84E-07
Os.8178.1.S1_at	LOC4350823	4.88	7.67E-05	4338883	Os05g0432200	12.27	3.39E-07
Os.39228.1.S1_at	LOC4348924	4.53	3.29E-04	4333794	Os03g0694000	11.74	3.77E-07
Os.11266.1.S1_at	LOC4335515	4.52	1.64E-05	4338137	Os05g0223200	11.00	3.07E-06
Os.6157.1.S1_at	LOC4324570	4.51	2.28E-05	4345814	Os08g0473900	10.74	4.40E-06
Os.11193.1.S1_at	LOC4334183	4.49	3.72E-07	4341420	Os06g0592500	10.70	1.05E-07
Os.54698.1.S1_at	LOC4333962	4.35	3.77E-05	4332361	Os03g0267000	10.55	1.00E-07
Os.36496.1.S1_at	LOC4344693	4.27	1.23E-05	4345814	Os08g0473900	10.48	9.12E-07
Os.9805.1.S1_at	LOC4351985	4.18	9.08E-06	4340484	Os06g0215500	10.48	1.12E-06
Os.2612.1.S1_at	LOC4349190	4.13	1.04E-05	4341420	Os06g0592500	10.46	8.03E-08

### 3.2 Obtaining the intersection of DEGs

**Fig. 1:**
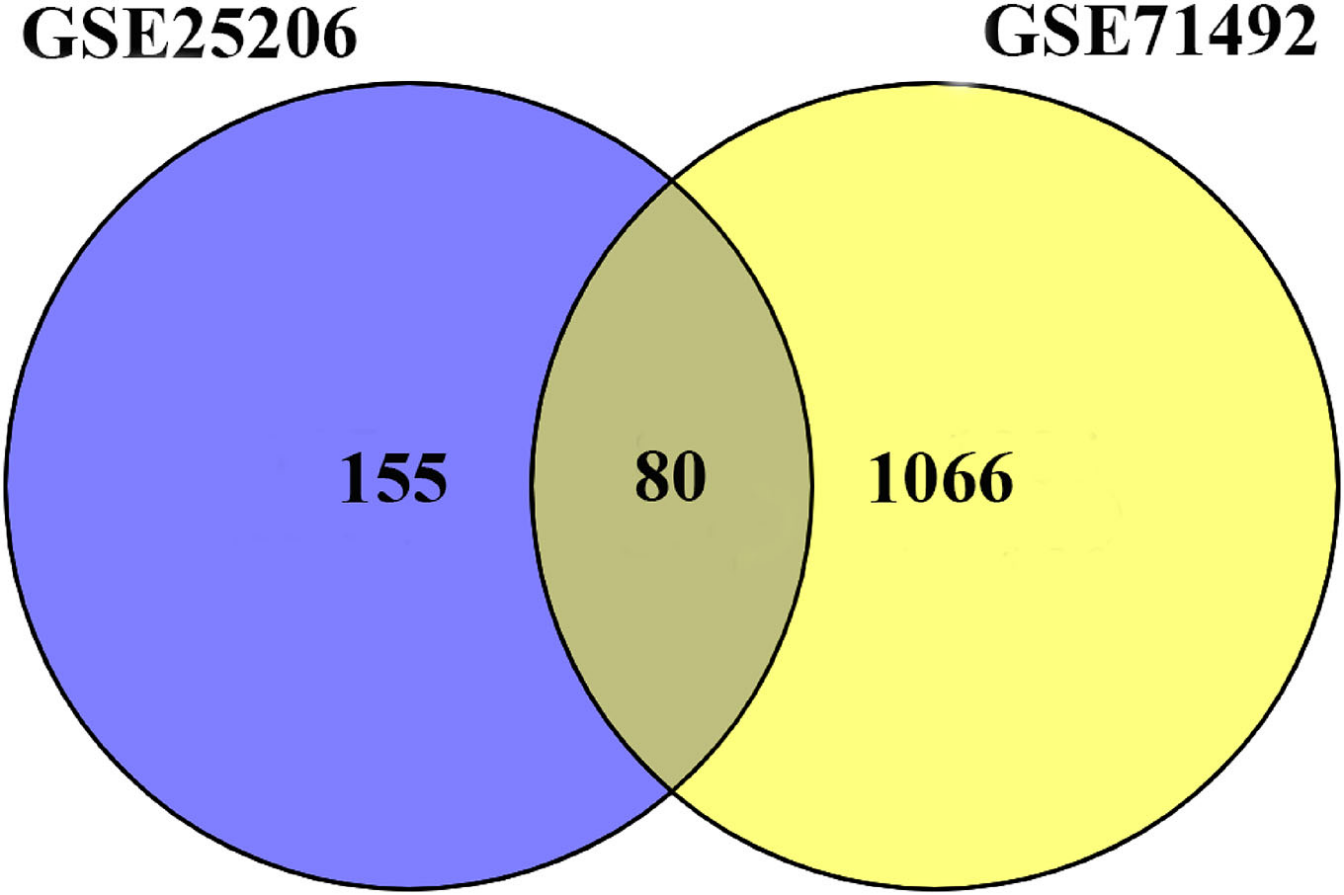
Intersection of differentially expressed genes in the GSE25206 and GSE71492 dataset.

In order to filter out common genes that were dysregulated both in the GSE25206 and GSE71492 datasets, the intersection of the DEGs in the two gene expression profiles were screened out using Venn analysis. As shown in Fig. 1, there were 80 DEGs including sixty-three up-regulated genes and seventeen down-regulated genes at the intersections, respectively.

### 3.3 Functional and pathway enrichment analysis of the intersection of DEGs

**Fig. 2:**
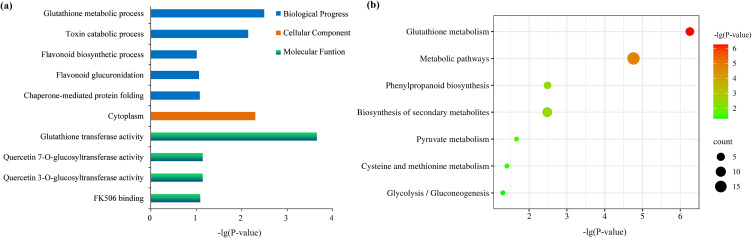
Functional annotation and pathway enrichment of differential expression genes related to arsenic stress in rice root. (a) GO enrichment analysis. The longitudinal axis represents GO terms that differential expression genes significantly enriched. (b) KEGG pathway analysis. The longitudinal axis represents pathways that differential expression genes significantly enriched. The circle represents the gene number enriched in each pathway with the larger circle for more genes.

GO function and KEGG pathway enrichment analysis were performed to investigate the functions of the common DEGs originated from GSE25206 and GSE71492 with a cut-off of P-value <0.05. GO enrichment analysis indicated that these common DEGs of GSE25206 and GSE71492 were clustered into ten GO terms, mainly including glutathione transferase activity, glutathione metabolic process, cytoplasm, toxin catabolic process, etc. (Fig. 2a). KEGG pathway enrichment analysis showed that these common DEGs of GSE25206 and GSE71492 were mainly enriched in seven pathways, mainly including glutathione metabolism, metabolic pathways, phenylpropanoid biosynthesis, biosynthesis of secondary metabolites, pyruvate metabolism, cysteine and methionine metabolism, glycolysis/gluconeogenesis (Fig. 2b).

### 3.4 Construction of PPI network and hub gene analysis

**Fig. 3:**
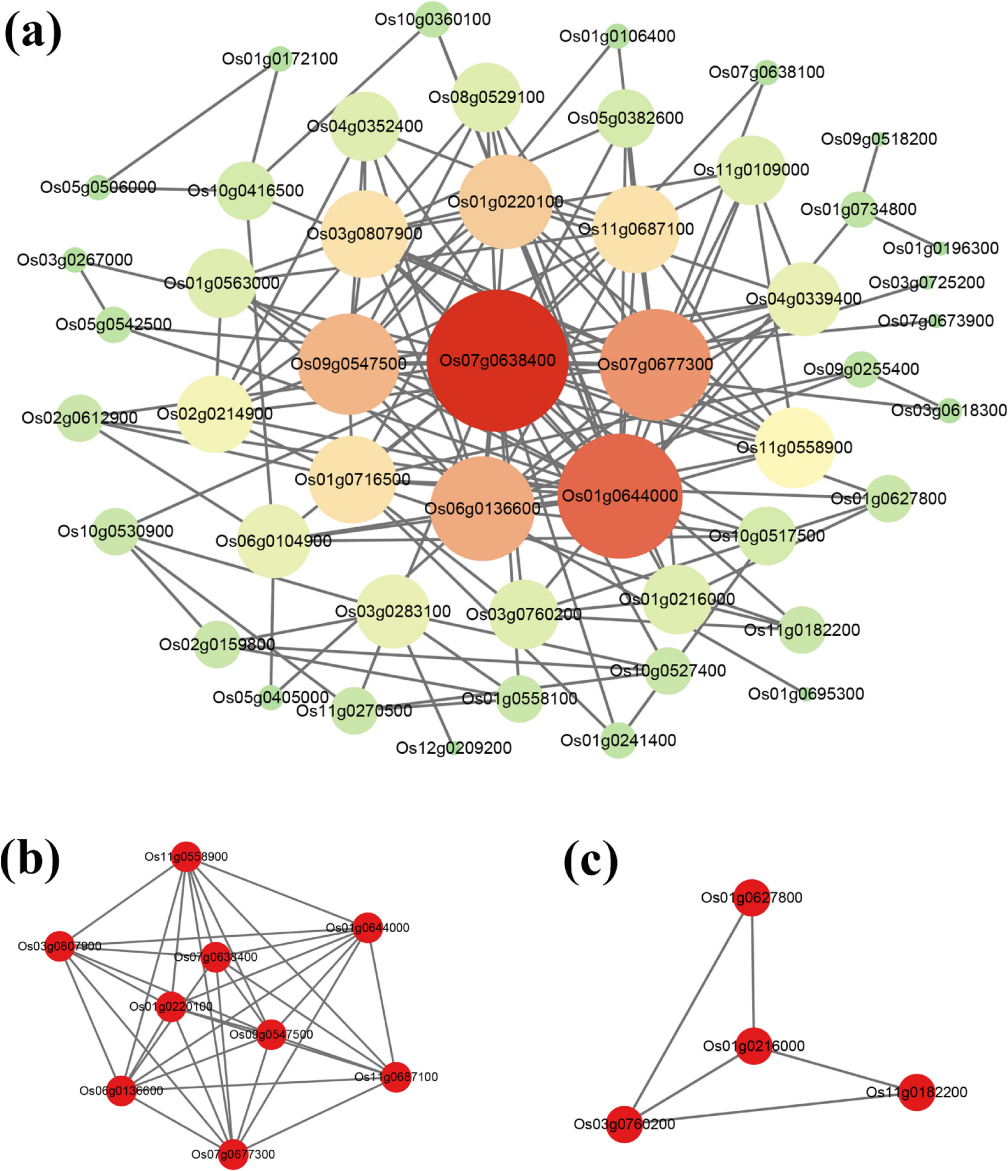
Construct a protein-protein interaction network of differential expression genes. (a) The protein-protein network of identified differentially expressed genes. The circle represents the protein node and circle size represents the degree. (b) and (c) were the top two modules identified from the protein-protein interaction network based on the degree of importance. (b) was Module 1 and (c) was Module 2.

In order to screen out the key genes for arsenic stress in rice, the PPI network of DEGs was constructed by using STRING. Then, a PPI network including 49 nodes and 150 edge interactions was obtained and visualized by Cytoscape (Fig. 3a). In addition, the MCODE plug-in of Cytoscape was applied to screen out the crucial modules of the PPI network. Then, two most significant modules were identified from the PPI network according to the degree of importance (Fig. 3b, c). Module 1 contains 9 nodes and 34 edges, and Module 2 contains 4 nodes and 5 edges. Subsequently, GO function and KEGG pathway enrichment analysis of genes in the two modules were carried out, respectively. The genes in module 1 were mainly enriched in cytosol and peroxidase activity GO terms and three pathways including phenylpropanoid biosynthesis, metabolic pathways and biosynthesis of secondary metabolites. The genes in module 2 were mainly enriched in four GO terms including oxidoreductase activity, monooxygenase activity, iron ion binding and heme binding (Table 2).

**Table 2:** GO function and KEGG pathway enrichment analysis of genes in Module 1 and Module 2

**Module 1**			

**Term**	**Description**	**Count**	**P-value**
GO:0005829	cytosol	3	0.019356
GO:0004601	peroxidase activity	2	0.046648
osa00940	Phenylpropanoid biosynthesis	2	0.001972
osa01100	Metabolic pathways	4	0.002740
osa01110	Biosynthesis of secondary metabolites	3	0.003842

**Module 2**			

**Term**	**Description**	**Count**	**P-value**

GO:0016705	oxidoreductase activity, acting on paired donors, with incorporation or reduction of molecular oxygen	2	0.02280
GO:0004497	monooxygenase activity	2	0.04077
GO:0005506	iron ion binding	2	0.04219
GO:0020037	heme binding	2	0.04816

Furthermore, important nodes and hub genes in the PPI network were predicted and explored using the Cytoscape-cytoHubba plug-in. There were eleven topological algorithms in CytoHubba, including degree, edge percolated component (EPC), maximum neighborhood component (MNC), density of maximum neighborhood component (DMNC), maximal clique centrality (MCC) and six centralities (bottleneck, eccentricity, closeness, radiality, betweenness and stress) based on shortest paths [37]. According to the methods of Baowei Xu [38] and Zhengfei Ma [39], in the present study, the hub genes were identified using nine topological analysis methods: degree, EPC, MNC, MCC, bottleneck, closeness, radiality, betweenness and stress algorithms, and the top 10 hub genes of each algorithm were shown in Table 3. The top 5 hub nodes in most rank methods were identified as hub genes that could be the best candidates associated with arsenic stress in rice root, including *Os01g0644000*, *PRDX6* (*Os07g0638400*), *PRX112* (*Os07g0677300*), *ENO1*(*Os06g0136600*), *LOGL9 *(*Os09g0547500*), which all belong to Module 1 (Fig. 3b).

**Table 3:** The hub genes (top 10) ranked in cytoHubba using different topological analysis methods.

**Category**	**Rank methods in CytoHubba**								

	**Degree**	**EPC**	**MNC**	**MCC**	**BottleNeck**	**Closeness**	**Radiality**	**Betweenness**	**Stress**
1	**Os07g0638400**	**Os07g0638400**	**Os01g0644000**	**Os01g0644000**	**Os07g0638400**	**Os07g0638400**	**Os07g0638400**	**Os07g0638400**	**Os07g0638400**
2	**Os01g0644000**	**Os01g0644000**	**Os07g0638400**	**Os09g0547500**	**Os07g0677300**	**Os01g0644000**	**Os01g0644000**	**Os07g0677300**	**Os07g0677300**
3	**Os07g0677300**	**Os07g0677300**	**Os07g0677300**	**Os07g0677300**	**Os06g0136600**	**Os07g0677300**	**Os07g0677300**	Os03g0760200	**Os01g0644000**
4	**Os06g0136600**	**Os09g0547500**	**Os09g0547500**	**Os06g0136600**	Os06g0104900	**Os06g0136600**	**Os06g0136600**	**Os01g0644000**	**Os06g0136600**
5	**Os09g0547500**	**Os06g0136600**	**Os06g0136600**	Os11g0558900	**Os01g0644000**	**Os09g0547500**	**Os09g0547500**	**Os06g0136600**	Os03g0760200
6	Os01g0220100	Os01g0220100	Os01g0220100	Os01g0220100	Os03g0760200	Os03g0807900	Os03g0807900	Os10g0416500	Os03g0283100
7	Os03g0807900	Os03g0807900	Os03g0807900	**Os07g0638400**	Os10g0416500	Os11g0687100	Os11g0687100	Os01g0734800	Os10g0416500
8	Os11g0687100	Os11g0687100	Os11g0687100	Os03g0807900	Os10g0527400	Os01g0220100	Os11g0558900	Os03g0283100	**Os09g0547500**
9	Os01g0716500	Os11g0558900	Os01g0716500	Os11g0687100	Os01g0734800	Os01g0716500	Os01g0716500	Os06g0104900	Os01g0716500
10	Os11g0558900	Os01g0716500	Os11g0558900	Os04g0339400	Os03g0807900	Os11g0558900	Os04g0339400	Os01g0716500	Os01g0734800

## 4. Discussion

Arsenic can be enriched in rice seedlings and have a negative effect on the growth and development of rice. But, the molecular mechanisms of rice in response to arsenic stress remain unclear. Understanding the molecular mechanisms might provide new ideas to reduce arsenic accumulation in rice. In the present paper, datasets that involving arsenic-treated rice root and the “study type” that was restricted to “Expression profiling by array” were selected and downloaded. DEGs predicted to play crucial roles in rice root in response to arsenic stress were screened out and the common DEGs of different datasets were obtained. Then, the biological function and pathway enrichment analysis of these DEGs were performed. Additionally, the corresponding PPI network and MCODE, CytoHubba analysis of DEGs was constructed and hub genes that might play important roles in rice response to arsenic stress were screened out.

GO analysis were used to classify the functions of the common DEGs originated from GSE25206 and GSE71492 dataset in biological progress, cellular component and molecular function. GO annotation revealed that the top 5 terms in biological process were glutathione metabolic process, toxin catabolic process, flavonoid biosynthetic process, flavonoid glucuronidation, and chaperone-mediated protein folding. In cellular component, it mainly affects the formation of cytoplasm. Furthermore, in molecular function, it mainly affects glutathione transferase activity, quercetin 7-O-glucosyltransferase activity, quercetin 3-O-glucosyltransferase activity, and FK506 binding (Fig. 2a). In addition, we used KEGG pathway analysis to further clarify the enriched metabolic pathways of DEGs. KEGG pathway analysis indicated that DEGs were significantly enriched in seven pathways, including glutathione metabolism, metabolic pathways, phenylpropanoid biosynthesis, biosynthesis of secondary metabolites, pyruvate metabolism, cysteine and methionine metabolism, glycolysis/gluconeogenesis (Fig. 2b).

The interactions between DEG-encoded proteins were explored using PPI network analysis. The PPI networks of the 80 common DEGs from the GSE25206 and GSE71492 dataset were constructed using the STRING database online tool with a combined score of more than 0.4 was defined as significant. The Cytoscape-cytoHubba plug-in was applied to predicate and explore the important nodes and hub DEGs in the PPI network. CytoHubba contains 11 algorithms. Finally, we chose the degree, EPC, MNC, MCC, bottleneck, closeness, radiality, betweenness and stress algorithms and screened out the five most important DEGs from PPI network analysis, including *Os01g0644000*, *PRDX6* (*Os07g0638400*), *PRX112* (*Os07g0677300*), *ENO1*(*Os06g0136600*), *LOGL9* (*Os09g0547500*).

*Os01g0644000* is a twin-arginine translocation (Tat) pathway signal domain containing gene. The Tat system, found in the cytoplasmic membranes of many eubacteria, some archaea, and the chloroplasts and mitochondria of plants, has the highly unusual property of transporting fully folded proteins [40], such as peroxidase [41]. The Tat pathway is required for many important cellular processes, including energy metabolism (Tat can translocate cofactor-containing proteins across biological membranes), cell division, cell motility, quorum sensing, heavy metal resistance [42], iron acquisition, and biofilm formation [43]. Furthermore, in response to abiotic stress, such as drought, *Os01g0644000* expression was up-regulated after drought stress [44]. Similarly, in the present study, *Os01g0644000* expression was up-regulated after arsenic stress in both the GSE25206 and GSE71492 datasets. It can be inferred that *Os01g0644000* might be the key gene in response to abiotic stress.

In addition, we found two genes related to peroxidase, *PRDX6*(*Os07g0638400*) and *PRX112* (*Os07g0677300*). *Os07g0638400*, named 1-Cys peroxiredoxin B, belongs to the peroxiredoxin family, Prx6 subfamily. Heavy metal-induced oxidative stress was one of the mechanisms of toxicity caused by heavy metals. Reports showed that the levels of intracellular reactive oxygen species, such as malondialdehyde and hydrogen peroxide, increased significantly when expose to heavy metal, while the activities of all antioxidant enzymes were decreased, such as superoxidase, peroxidase, and glutathione predominately [45]. Meanwhile, reports showed that some heavy metal antagonists, such as melatonin and sodium nitroprusside, could significantly improve peroxidase activities to cope with the stress of heavy metals in plants [46, 47]. *PRDX6* has been reported to be one of the best candidates associated with anaerobic germination tolerance [48]. *PRX112* has been reported to participate in the rice seedlings in response to the stress from H_2_O_2_ [49]. Presently, *PRDX6* expressions were up-regulated, while *PRX112* were down-regulated after arsenic stress in both the GSE25206 and GSE71492 datasets, indicating the different response strategies of the rice antioxidant system in response to arsenic stress.

Besides, we found a glycolysis-related gene *ENO1*(*Os06g0136600*), which is named enolase-1 and involved in phosphorylase reactions in glycolytic pathways. Presently, *ENO1* was up-regulated both in the GSE25206 and GSE71492 datasets after arsenic stress. This behavior is consistent with previous reports of rice under stress conditions. Reports showed that the *ENO1* involved in rice responds to abiotic stress. For example, *ENO1* was up-regulated significantly in cold-tolerance *Japonica* rice cultivars compared to the wild type [50]. In addition, the up-regulation of *ENO1* was also related to the biosynthesis of rice allelochemicals to increase the allelopathic potential of rice accessions [51].

Currently, *LOGL9* (*Os09g0547500*) were up-regulated in both the GSE25206 and GSE71492 datasets after arsenic stress. The *LOGL9* gene product was annotated as a lysine decarboxylase-like protein. Reports showed that *LOGL9* was related to the response of rice to oxidative stress. A highly oxidative stress-tolerant *japonica* rice line was isolated and found that *LOGL9* mutation induced significantly lower cellular levels of reactive oxygen species than those in the wild-type rice after exposure to oxidative, high salt and acid stresses [52].

Besides arsenic, rice is also highly efficient in cadmium accumulation and soil-to-rice transfer [53]. In order to alleviate the pollution of cadmium and arsenic in rice simultaneously, researchers have made many explorations by means of genetic engineering. Rice has two phytochelatin synthases, OsPCS1 and OsPCS2. Report showed that the loss of function of *OsPCS1*(*Os05g0415200*) in node I could lead to high arsenic concentration in rice grains, while *OsPCS2* (*Os06g0102300*) had little effect. However, OsPCS2 was more sensitive to activation by cadmium than by arsenic [27]. In the present paper, there is no sufficient evidence to show that the five arsenic-related genes in rice roots screened out have significant effects on cadmium accumulation in grains. Correspondingly, few specific genes in rice related to both cadmium and arsenic metabolism had been obtained, which might be linked to the different metabolic pathways of cadmium and arsenic in rice, especially the uptake and transport pathways. Cadmium is transported into the rice root cell mainly by carrier protein for essential metals, such as manganese transporter OsNramp5, iron transporters (OsNramp1, OsIRT1 and OsIRT2), and zinc transporters (OsZIP1 and OsZIP3) [54]. Silicon transporter OsLsi1 is the main constituent for trivalent arsenic uptake and accumulation in rice, and phosphate transporter OsPT8 has a high affinity for pentavalent arsenic [55].

## 5. Conclusions

In conclusion, a preliminary investigation of the molecular mechanisms of rice root in response to arsenic stress were performed in the present study. All related gene expression profiles were downloaded and common DEGs were screened out. DEGs functions were then explored by GO function and KEGG pathway enrichment analysis. Subsequently, five key hub genes that was predicted to play crucial roles in rice root in response to arsenic stress have been obtained using PPI analysis, including *Os01g0644000*, *PRDX6* (*Os07g0638400*), *PRX112* (*Os07g0677300*), *ENO1*(*Os06g0136600*), *LOGL9* (*Os09g0547500*). However, there are some shortcomings in the present study. The hub genes obtained from the public database by bioinformatics analysis has not been verified by experiments. In the next step, molecular biological experiments are required to confirm the function of the identified genes in rice root responses to arsenic stress.

## Abbreviations

GEO: Gene Expression Omnibus
DEGs: Differentially expressed genes
GO: Gene Ontology
KEGG: Kyoto Encyclopedia of Genes and Genomes
PPI: Protein-protein interactions
MCODE: Molecular complex detection algorithm
IARC: International Agency for Research on Cancer
BP: Biological process
CC: Cellular component
MF: Molecular function
STRING: Search Tool for the Retrieval of Interacting Genes/Proteins
EPC: Edge percolated component
MNC: Maximum neighborhood component
DMNC: Density of maximum neighborhood component
MCC: Maximal clique centrality
Tat: Twin-arginine translocation

## Declarations

### Availability of data and materials

The expression profiles datasets used in this study are publicly available at GEO database (https://www.ncbi.nlm.nih.gov/geo/): GSE25206, GSE99083, and GSE71492.
